# Supplementary material for: Effects of Pegylated Interferon Alpha and Ribavirin (pegIFN-α/RBV) Therapeutic Approach on Regulatory T Cells in HCV-Monoinfected and HCV/HIV-Coinfected Patients
Source: Viruses. 2021 Jul 25;13(8):1448. doi: 10.3390/v13081448 (PMC8402834; doi:10.3390/v13081448)
Supplement: Supplementary file 1 [file viruses-13-01448-s001.zip › viruses-1283654-supplementary.pdf]

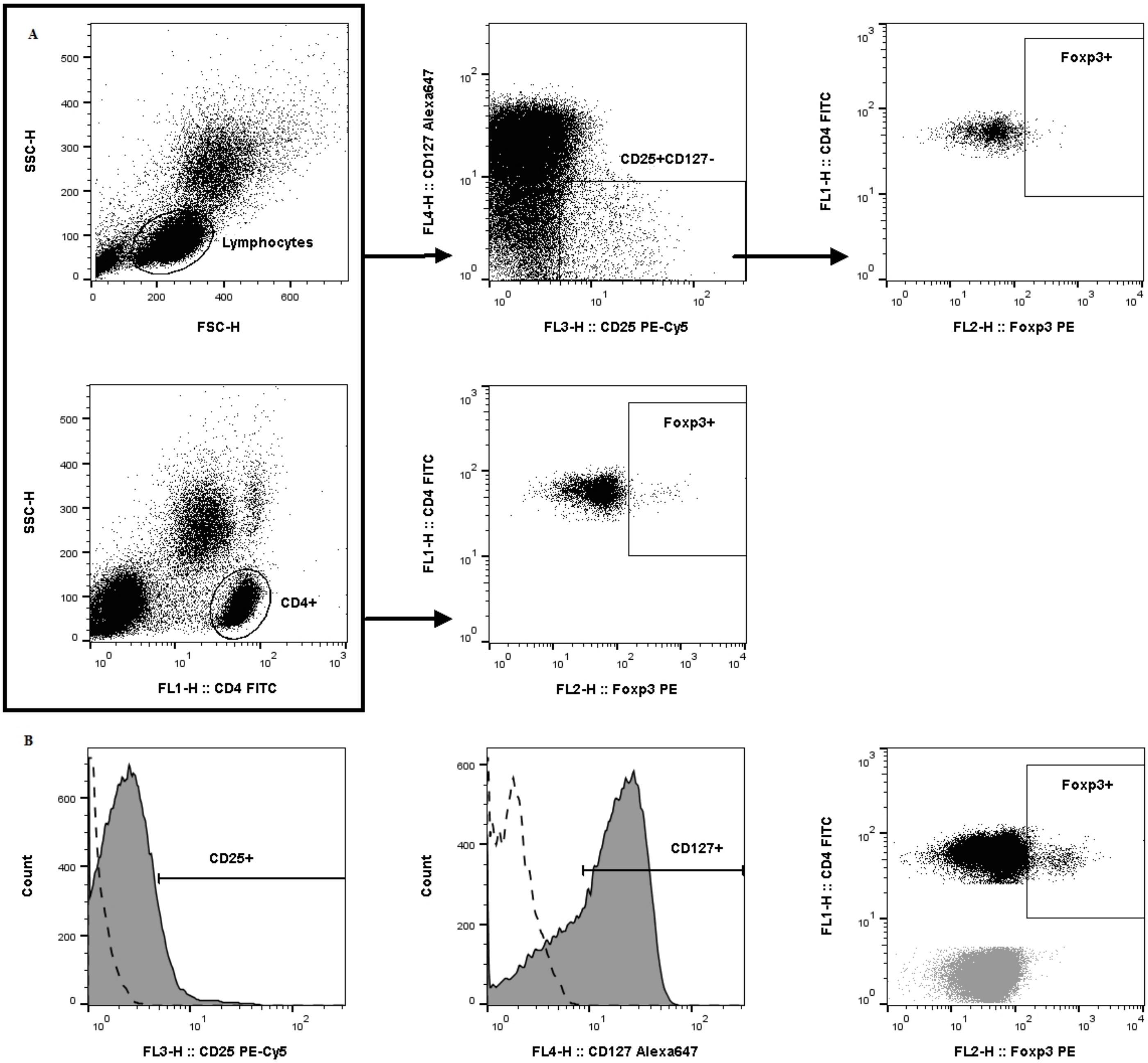

Figure S1. Gating strategy of regulatory T cells (Treg). Foxp3+ Tregs were evaluated on the basis of gating Foxp3-positive cells within CD4+ lymphocytes and CD25+CD127- cells (A). Delineation of positive signals for CD25 PE-Cy5, CD127 Alexa647 and Foxp3 PE was based on FMO (fluorescence-minus-one) and negative population controls (B).

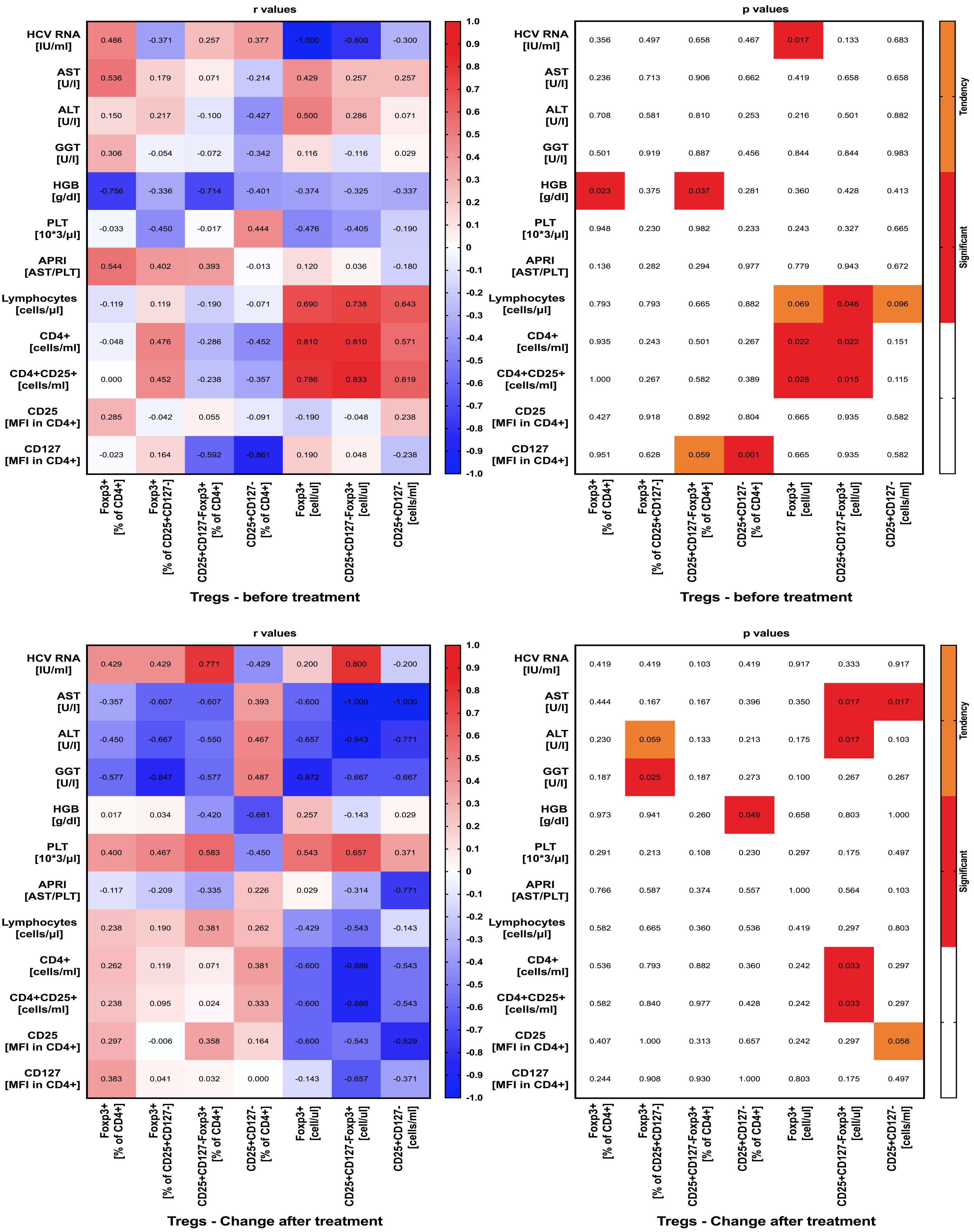

Figure S2. Graphical presentation of the regulatory T cells data correlations with virological, biochemical and immunological data in HCV-infected patients prior treatment application. Treg-related parameters before (upper graphs) and after treatment (% of after versus before; lower graphs) were analyzed. Statistically significant correlations were bolded in the table.

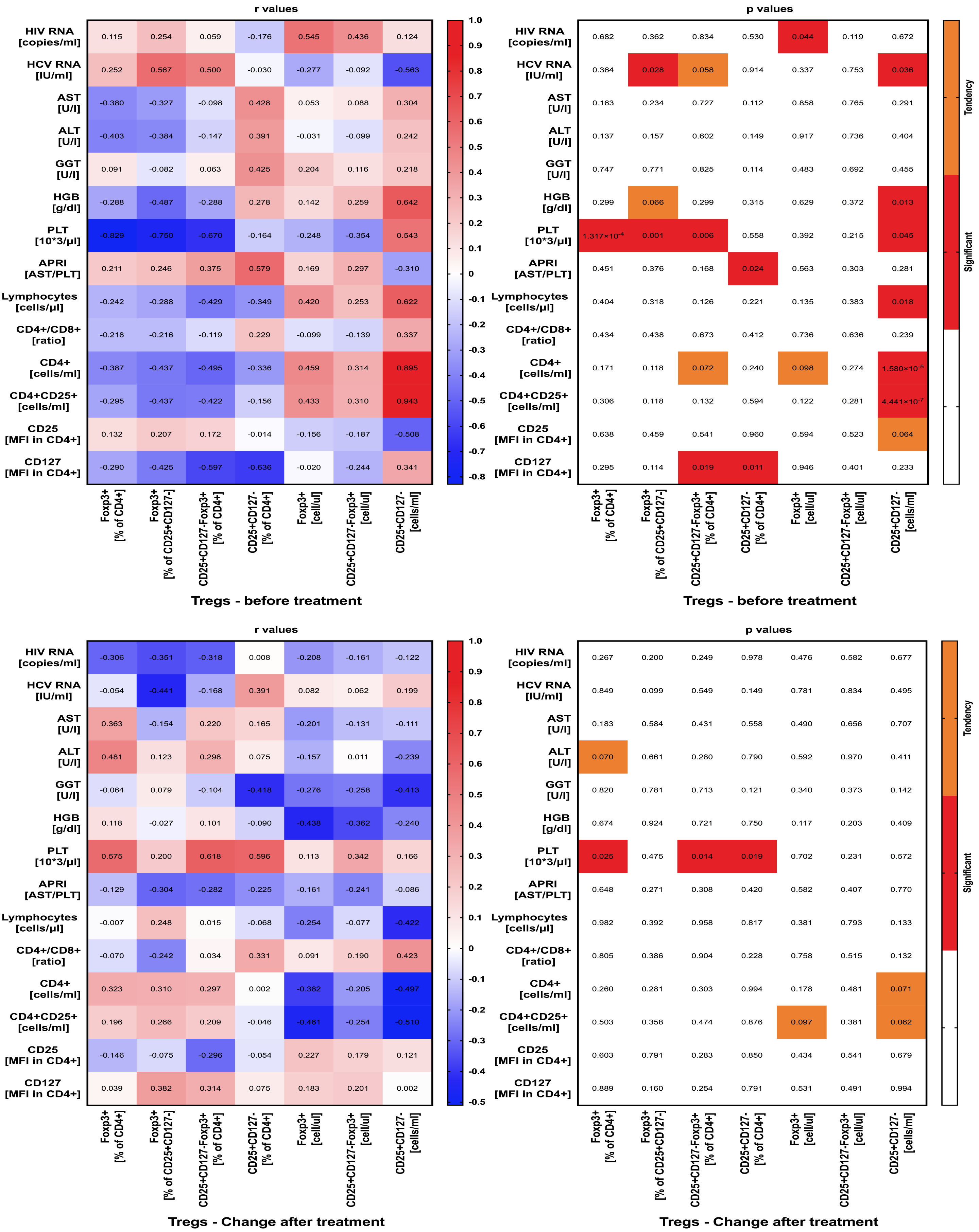

Figure S3. Graphical presentation of the regulatory T cells data correlations with virological, biochemical and immunological data in HCV/HIV-coinfected patients prior to treatment application. Treg-related parameters before (upper graphs) and after treatment (% of after versus before; lower graphs) were analyzed. Statistically significant correlations were bolded in the table.

	Healthy subjects	HCV-infected subjects	HCV/HIV-coinfected subjects
**Gender [male/female]**	10/8	6/5	11/5
**Age [years]**	37 [32; 46.5]	41 [28; 50]	31 [29; 33.5]
**Leukocytes [cells/µl]**	5890 [5430; 7140]	4410 [3228; 6300]	4610 [4148; 6108]
**Neutrophils [cells/µl]**	2940 [2415; 4235]	2185 [1955; 3145]	2470 [1520; 2790]
**Lymphocytes [cells/µl]**	1780 [1628; 2495]	1475 [808; 2040]	1730 [1300; 2130]
**Monocytes [cells/µl]**	610 [525; 760]	445 [395; 570]	510 [420; 610]
**Eosinophils [cells/µl]**	130 [85; 145]	65 [30; 137.5]	100 [60; 170]
**Basophils [cells/µl]**	30 [20; 45]	20 [10; 20]	20 [10; 50]
**Hemoglobin [g/dl]**	13.9 [13.4; 15.5]	13.9 [13.6; 15.65]	14.3 [13.55; 14.95]
**Platelets (PLT) [10^3/µl]**	211 [169.5; 239]	200 [156; 213.5]	159.5 [121; 219]
**AST [U/l]**		32 [25; 42]	51 [43; 79]
**APRI (AST/PLT Ratio Index)**		0.35 (0.10; 0.58)	0.85 (0.63; 1.10)
**ALT [U/l]**		47 [27; 80]	83.5 [48.25; 138.8]
**GGT [U/l]**		71 [20; 88]	65.5 [36.5; 145]
**HCV RNA [IU/ml]**		17100 [2875; 35580]	812 [264.5; 8423]
**HIV RNA [copies/ml]**			39 [39; 47.25]
**CD4+ lymphocytes [cells/µl]**			480 [394.5; 526]
**CD8+ lymphocytes [cells/µl]**			617.5 [439; 919.3]
**CD4+/CD8+ [ratio]**			0.78 [0.57; 1.06]

Table S1. Presentation of virological, biochemical and immunological data of healthy subjects, and HCV-infected and HCV/HIV-coinfected patients before treatment. Data presented as median values with 25th and 75th percentile in the brackets.

	Healthy subjects	HCV-infected patients before treatment	HCV-infected patients after treatment	HCV/HIV-infected patients before treatment	HCV/HIV-infected patients after treatment
**Foxp3+ [% of CD4+]**	1.86 (1.39; 2.46)	2.34 (2.02; 4.40)	**4.33**[d] **(3.10; 7.17)**	3.02 (2.11; 4.13)	**2.92**[b] **(2.04; 3.87)**
**Foxp3+ [% of CD4+ CD25+CD127-]**	3.93 (2.46; 7.31)	5.58 (5.14; 7.35)	11.80 (7.17; 20.80)	6.33 (4.04; 9.47)	6.98 (4.75; 12.30)
**CD25+CD127-Foxp3+ [% of CD4+]**	0.25 (0.10; 0.66)	0.72 (0.50; 1.12)	0.96 (0.76; 1.90)	0.84 (0.48; 1.44)	0.85 (0.53; 1.10)
**CD25+CD127- [% of CD4+]**	6.86 (3.80; 14.35)	12.20 (8.52; 14.50)	12.60 (6.46; 15.30)	12.90 (9.15; 17.40)	12.20 (7.21; 16.00)
**CD4+Foxp3+ [cells/ µl]**	7231 (6665; 7431)	7725 (4061; 12895)	**7956**[d] **(3929; 15008)**	5494 (4097; 9864)	**3619**[b] **(2492; 4802)**
**CD4+CD25+CD127- Foxp3+ [cells/ µl]**	439 (263; 577)	2245 (1156; 3841)	1443 (1140; 3921)	1422 (1156; 3183)	1076 (944; 1490)
**CD4+CD25+CD127- [10^3cells/ µl]**	17.79 (10.82; 18.06)	24.14 (19.85; 44.30)	13.98 (8.79; 33.21)	26.76 (18.59; 35.95)	13.58 (8.18; 29.14)
**CD4+ [10^3cells/ µl]**	412.39 (391.86; 501.12)	228.18 (180.57; 361.75)	145.27 (110.96; 154.44)	283.90 (123.03; 352.22)	138.27 (111.65; 202.92)
**CD25+ [% of CD4+]**	17.60 (15.10; 22.75)	20.30 (15.30; 22.70)	18.50 (15.60; 23.60)	24.00 (20.50; 27.20)	22.50 (18.10; 26.50)
**CD25 [MFI in CD4+]**	2.91 (2.68; 3.08)	3.02 (2.83; 3.91)	3.30 (2.97; 4.59)	3.04 (2.80; 3.43)	3.23 (2.94; 3.72)
**CD127+ [% of CD4+]**	55.60 (36.05; 85.25)	24.70 (18.70; 56.80)	38.90 (22.20; 46.80)	40.70 (23.30; 51.90)	46.40 (25.10; 54.70)
**CD127 [MFI in CD4+]**	4.67 (2.77; 14.85)	2.35 (1.96; 5.05)	2.88 (2.21; 3.61)	3.04 (2.22; 3.95)	3.31 (2.12; 4.47)

Table S2. Tabular presentation of regulatory T cells, T cell activation (CD25) and development-associated protein (CD127) results in healthy subjects, HCV-infected and HCV/HIV-coinfected patients (data presented as median values with 25th and 75th percentile in the brackets). Bolded values indicate differences between HCV- and HCV/HIV-infected groups (significantly compared to: a – HCV-infected before treatment, b – HCV-infected after treatment, c – HCV/HIV-infected before treatment, d – HCV/HIV-infected after treatment).
